# Automated analysis of cell migration and nuclear envelope rupture in confined environments

**DOI:** 10.1371/journal.pone.0195664

**Published:** 2018-04-12

**Authors:** Juniper J. Elacqua, Alexandra L. McGregor, Jan Lammerding

**Affiliations:** Meinig School of Biomedical Engineering, Weill Institute for Cell and Molecular Biology, Cornell University, Ithaca, NY, United States of America; Medical College of Wisconsin, UNITED STATES

## Abstract

Recent *in vitro* and *in vivo* studies have highlighted the importance of the cell nucleus in governing migration through confined environments. Microfluidic devices that mimic the narrow interstitial spaces of tissues have emerged as important tools to study cellular dynamics during confined migration, including the consequences of nuclear deformation and nuclear envelope rupture. However, while image acquisition can be automated on motorized microscopes, the analysis of the corresponding time-lapse sequences for nuclear transit through the pores and events such as nuclear envelope rupture currently requires manual analysis. In addition to being highly time-consuming, such manual analysis is susceptible to person-to-person variability. Studies that compare large numbers of cell types and conditions therefore require automated image analysis to achieve sufficiently high throughput. Here, we present an automated image analysis program to register microfluidic constrictions and perform image segmentation to detect individual cell nuclei. The MATLAB program tracks nuclear migration over time and records constriction-transit events, transit times, transit success rates, and nuclear envelope rupture. Such automation reduces the time required to analyze migration experiments from weeks to hours, and removes the variability that arises from different human analysts. Comparison with manual analysis confirmed that both constriction transit and nuclear envelope rupture were detected correctly and reliably, and the automated analysis results closely matched a manual analysis gold standard. Applying the program to specific biological examples, we demonstrate its ability to detect differences in nuclear transit time between cells with different levels of the nuclear envelope proteins lamin A/C, which govern nuclear deformability, and to detect an increase in nuclear envelope rupture duration in cells in which CHMP7, a protein involved in nuclear envelope repair, had been depleted. The program thus presents a versatile tool for the study of confined migration and its effect on the cell nucleus.

## Introduction

Cell migration is necessary for a number of important physiological processes including immune response, wound healing, and cancer metastasis. Cell migration is particularly important in the context of cancer metastasis, which is responsible for the vast majority of cancer-related deaths, including over 90% of breast cancer deaths [1]. For cancer cells to metastasize, they must first migrate away from the site of the primary tumor (invasion), enter blood or lymphatic vessels (intravasation) through which they are transported to distant parts of the body, and then exit the vessels (extravasation) and migrate to new sites, where they may grow into secondary tumors [1, 2]. The migration behavior of cancer cells is a good indicator of patient prognosis, as more migratory cells form metastases at higher rates. Preventing or reducing cancer cell migration could significantly improve cancer patient outcomes, and present a key step in reducing metastasis-related mortality.

During the processes of tissue invasion and intra- and extravasation, cancer cells have to squeeze through small spaces between other cells and within the extracellular matrix (ECM). Recent findings point to an important role of the cell nucleus in the migration of cells through such confined environments [3]. Deformation of the nucleus, which is the largest and stiffest cellular organelle, determines the ability of cells to pass through constrictions smaller than the nuclear cross-section [3–5]. Cells with less deformable nuclei take longer to pass through microscopic pores than cells with more deformable nuclei [6–8]. One of the primary determinants of nuclear deformability is the expression of lamins A and C, intermediate filament proteins that form a dense protein network (nuclear lamina) underneath the inner nuclear membrane [9, 10]. Intriguingly, expression of lamin A/C is decreased in many cancers [11–15], which could contribute to increased metastatic potential of tumor cells by facilitating both invasion and intra- and extravasation.

In addition to modulating transit efficiency through confined environments, migration through tight spaces places substantial physical stresses on the nucleus, which can lead to a transient loss of nuclear envelope (NE) integrity during interphase, referred to as NE rupture [16, 17]. NE rupture, which allows uncontrolled exchange of cytoplasmic and nuclear proteins, along with protrusion of chromatin into the cytoplasm, could result in increased genomic instability and promote cancer progression [18]. Cells can restore NE integrity using components of the endosomal sorting complexes required for transport-III (ESCRT-III) machinery [16, 17]. Inhibiting NE repair, when combined with inhibition of DNA damage repair pathways, results in substantially increased cell death after NE rupture [16, 17], pointing to potential treatment approaches to specifically target metastatic cancer cells.

**Fig 1:**
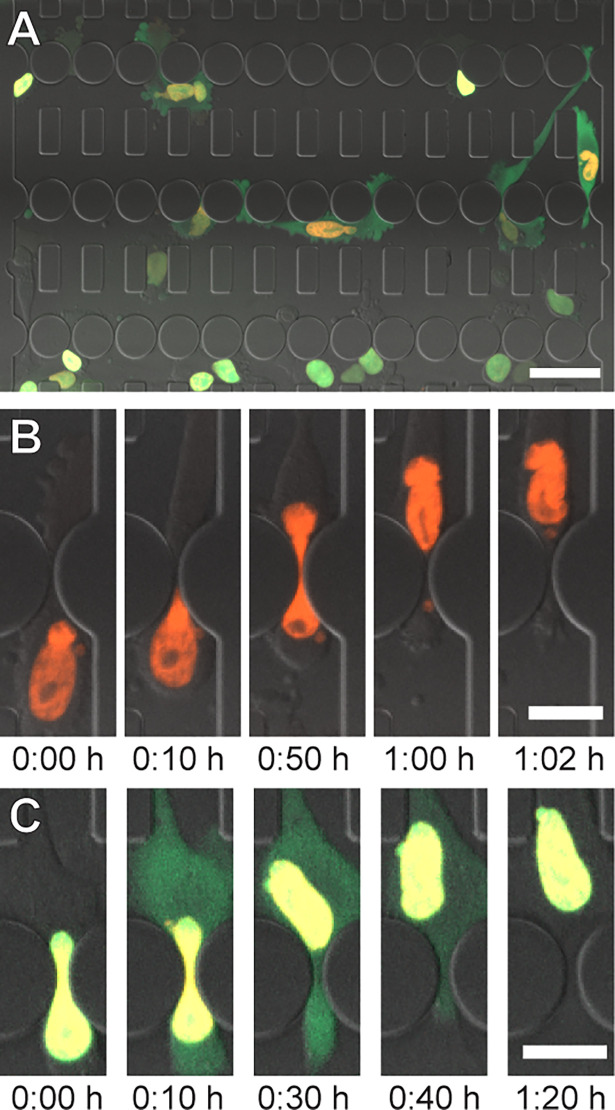
Cell migration through microfluidic constrictions. (**A**) Cells expressing NLS-GFP and H2B-tdTomato migrating through a microfluidic device. Scale bar: 50 μm. (**B**) Time series of a nucleus squeezing through a constriction. Scale: bar 20 μm. (**C**) Time series of a NE rupture event. NLS-GFP leaks into the cytoplasm upon NE rupture and is reimported into the nucleus as the NE is repaired. Scale bar: 20 μm.

These findings have motivated a rapidly growing interest in studying nuclear deformation and NE rupture, particularly during confined migration [10, 18–22]. Microfluidic devices with precisely defined constrictions that mimic interstitial spaces *in vivo* have emerged as powerful tools to study the role of nuclear deformation and NE rupture in cell migration [7, 16, 17, 20, 23–28]. Although the walls of such devices are more rigid than the *in vivo* spaces through which cells migrate, confined migration and NE rupture results obtained in these microfluidic devices closely match those obtained in collagen matrices and from intravital imaging studies [16, 17], and the devices enable time-lapse imaging of single-cell migration under precisely defined conditions. In such experiments, nuclei are often identified by fluorescently labeled DNA (e.g., staining with Hoechst 33342) or histones (e.g., expression of H2B-tdTomato). NE rupture events can be detected by monitoring the intracellular localization of a green fluorescent protein containing a nuclear localization sequence (NLS-GFP) [16, 17, 29]. NLS-GFP is normally contained within the nucleus but spills into the cytoplasm during NE rupture and is gradually re-imported into the nucleus upon NE repair (Fig 1). Time-lapse experiments using cancer cells typically cover 6 to 24 hours, with multi-color (fluorescence and transmitted light) images acquired every 2 to 10 minutes, resulting in large (>40 GB per experiment), multi-dimensional data sets that take several days to weeks to manually analyze. Such low throughput image analysis provides a substantial challenge when studying large sets of experimental conditions. Furthermore, manual analysis by different observers can add substantial variability to the experimental data.

To address these issues, we developed a MATLAB program to perform the image analysis in an automated, reproducible, and robust process. The program is capable of tracking individual cells/nuclei as they migrate through microfluidic constriction channels and compute transit times for individual constrictions. While primarily intended to study cell migration in confined environments, the program can also be used to study cells migrating on unconfined 2-D substrates. The progam can also reliably detect NE rupture events and their duration. The program automatically recognizes dividing cells, resulting in increased robustness and accuracy comparable to expert manual analysis, but with substantially increased efficiency.

## Automated analysis algorithm

### Overview

**Fig 2:**
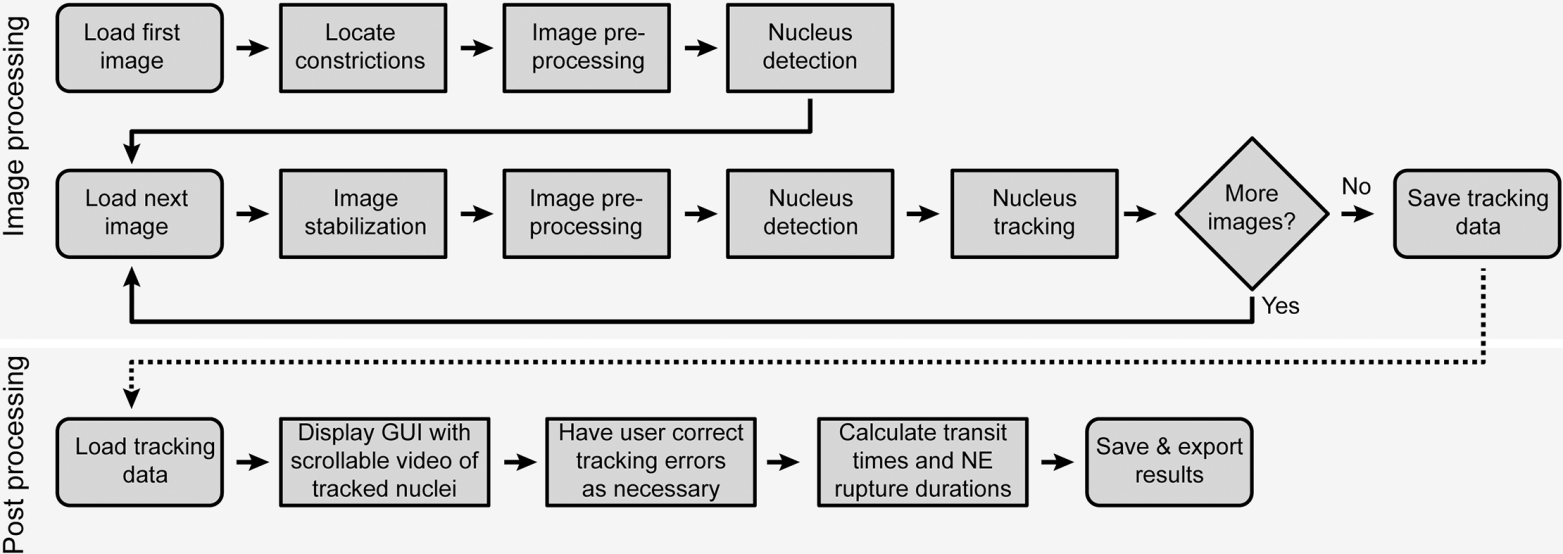
Flowchart of automated analysis steps. **S**teps the program takes when analyzing an image sequence are detailed, including image processing (top) and post-processing (bottom).

Automated image analysis begins by locating the constrictions in the first image (Fig 2). The image is then processed to reduce noise and to detect fluorescently labeled nuclei. For each subsequent image in the sequence, image stabilization is performed to account for small shifts in the field of view during image acquisition. Each image is then subject to the same processing as above to reduce noise and detect nuclei. Identified nuclei are tracked from the previous image to the current one. All nuclei are then observed for incidences of constriction passage and NE rupture. After the full sequence has been analyzed, the tracking results are exported to a spreadsheet and presented to the user for manual validation (Fig 2). The program was implemented in MATLAB 2016a and runs on all MATLAB supported platforms, version 2016a and newer. It can be downloaded at the following URL: https://github.com/Lammerding/MATLAB-CellTracking.

### Locating constrictions

Constriction location is performed by identifying the round pillars in the microfluidic devices that form the three rows of constrictions (Fig 1A and S1 Fig). This is accomplished by applying a circular Hough transform (a technique that identifies circles in an image) to a transmitted light image of the device. All images are then rotated to align the rows of constrictions horizontally. Virtual boundaries are defined at a specific distance above and below the constriction centerline to determine nucleus entry and exit for each row of constrictions. (S1 Fig). This approach can be adjusted for devices with different designs.

#### Image pre-processing and stabilization

To reduce the noise in the fluorescence images and enhance the contrast between the nuclear signals and the background, a 10 × 10 pixel Gaussian filter is applied to the images of the fluorescence channels. Image stabilization is then performed via normalized 2D cross-correlation between an image and its predecessor. The obtained spatial offset values are applied to the transmitted light as well as the fluorescence image channels.

### Nucleus detection

**Fig 3:**
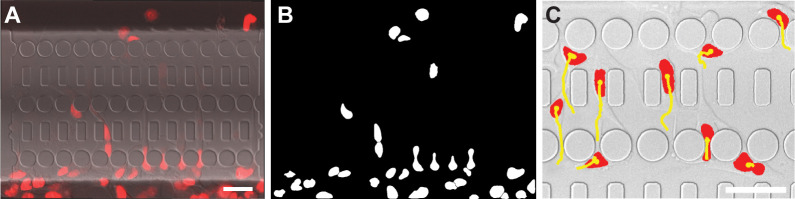
Examples of nuclear identification and tracking. (**A**) Merged image of the transmitted light and tdTomato channels. Nuclei (red) can be seen in the migration device. Scale bar: 50 μm. (**B**) Binarized version of red channel of image A. Each nucleus is identified as a separate object (white). (**C**) Example of tracking results. Nuclei (red) have been identified, and their centroid positions during migration are shown as yellow tracks. For clarity, tracks displayed here are limited to data for only the last six hours.

Nuclei are identified by binarizing images with a locally adaptive threshold based on their H2B-tdTomato signal and applying connected component analysis. Local thresholding, while more computationally expensive, provides better results than global thresholding, especially for unevenly illuminated images. To separate touching nuclei into distinct objects, further segmentation is necessary. Since nuclei are generally oval-shaped, the program uses watershed segmentation based of the distance transform of the identified nuclei. Watershedding segments an image based on “watershed lines”, which separate the image into different “catchment basins”. Figuratively, the image is treated like a topographic map, with image intensities representing the height. This means image areas containing low pixel intensity values are grouped together and separated from other groups if there are high pixel intensity values between them (the “watersheds”). The resulting regions are converted into binary images and further processed to distinguish single nuclei, which form a single catchment basin, from multiple touching nuclei, which form multiple basins separated by a watershed line. Additional imaging process steps are applied to prevent over-segmentation. A visual guide to this algorithm is provided in S2 Fig. Nuclei inside of constrictions are excluded from this segmentation since they take on a dumbbell shape (Fig 1B, 0:50 h) and otherwise may be incorrectly split into two objects. After image segmentation, identified objects are deleted if their properties, such as size and circularity, suggest that they are not nuclei (Fig 3A and 3B).

### Nucleus tracking

After nuclei have been identified, they are tracked over time by recording their centroid position at each time point. The following error function is applied to every possible pairing of a nucleus in one image and a nucleus in the following image:

$$E=distance^{2}+2*|\Delta fluorescent\;intensity|+2*|\Delta area|$$

This error function is related to the likelihood that two objects are the same nucleus and is based on the square of the distance between the centroids of the two objects. Since comparing only the distances between objects produces inaccurate results when multiple nuclei are in close proximity, the error function also includes the change in each object’s area and its average H2B-tdTomato fluorescent intensity. These values are expected to remain relatively constant over time for individual nuclei, but vary between different nuclei. The object from the previous time point and the object in the current time point that together have the lowest error function value are paired with one another and marked as unavailable for other pairings. Pairs can only be made if the centroids of the objects are within 40 μm of one another, which is the maximum distance a cell typically travels within the chosen time interval. If necessary, the user can later correct this during the manual validation and editing stage, but we found that such instances are rare. Object pairing and marking availability status continues until no further object pairings are available. Upon completion, time-resolved data for each identified nucleus include centroid position, bounding box, area, and fluorescence intensity, which can be displayed for each nucleus (Fig 3C) and used for further analysis.

### Detection of cell transit through constrictions

The passage of nuclei through constrictions is evaluated as follows: if the top of a nucleus’ bounding box is above the lower boundary of a constriction and the bottom of the nucleus’ bounding box is below the upper boundary of the constriction, the nucleus is considered to be attempting to pass through the constriction (Part A in S3 Fig). When an attempting nucleus moves completely above the upper bounding box, it is recorded as having successfully passed the constriction (Part B in S3 Fig). A nucleus that is attempting to traverse a constriction but then moves back out of the boundary of the constriction is recorded as failing to pass through the constriction (Part C in S3 Fig). Nuclei that only briefly (1 time point) attempt to enter a constriction are excluded from the analysis. Such instances can occur when a cell moves parallel to the row of constrictions and a part of the nucleus crosses the boundary of the constriction, without the cell attempting to pass through the constriction.

### NE rupture detection

**Fig 4:**
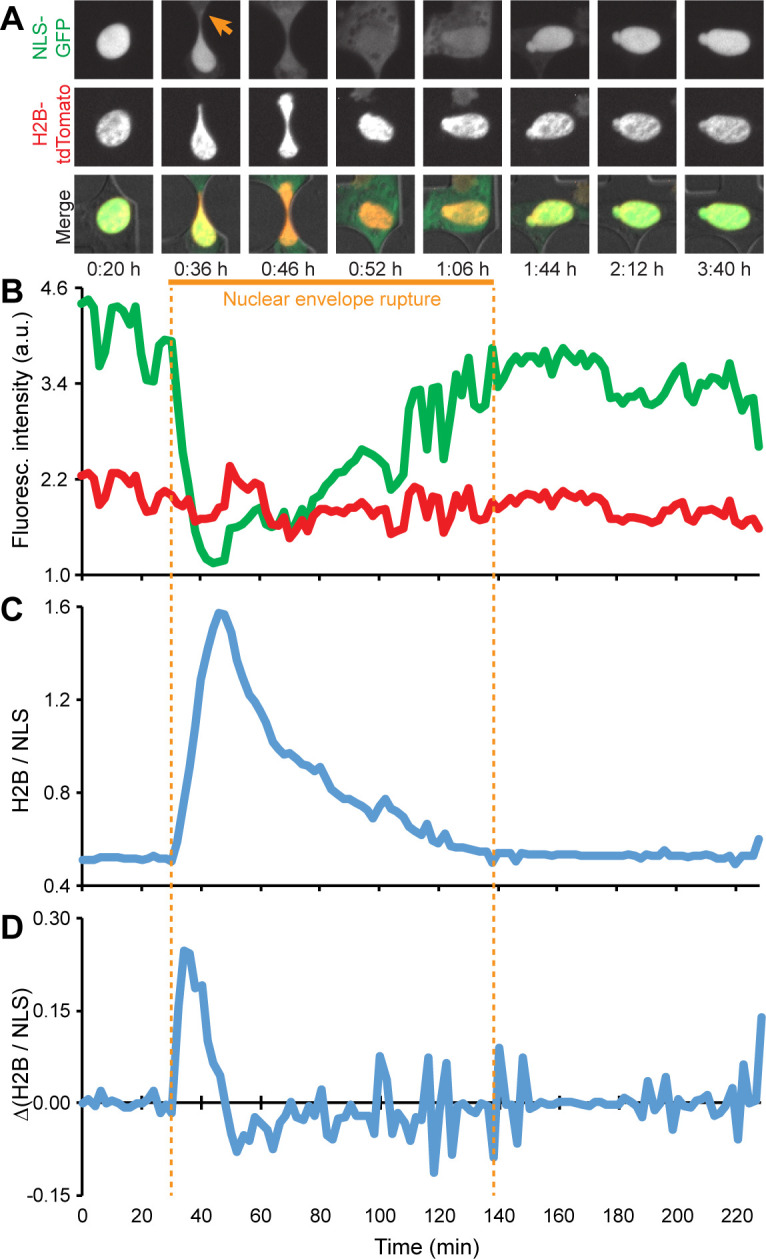
Detection of NE rupture. (**A**) During NE rupture (arrow), NLS-GFP (green) spreads throughout the cytoplasm, causing the nuclear NLS-GFP signal to lose intensity. In contrast, the H2B-tdTomato signal (red) remains approximately constant. (**B**) Normalizing these two signals against one another (H2B/ NLS) significantly reduces the effects of noise and allows for more accurate NE rupture detection. (**C**) Steep increases in the H2B/NLS ratio, which correspond to high values of Δ(H2B/NLS), plotted in (**D**), indicate the start of a NE rupture event. The data shown here are for a representative cell.

NE rupture is detected by monitoring the inverse ratio of the nuclear NLS-GFP signal to the H2B-tdTomato signal. Since the total amount of NLS-GFP per cell stays approximately constant over time, and NLS-GFP spills into the cytoplasm during NE rupture, the average NLS-GFP nuclear intensity decreases during NE rupture. In contrast, the H2B-tdTomato signal remains nearly constant, allowing for normalization to the H2B-tdTomato signal. The normalization accounts for variations due to photobleaching and other image acquisition effects. The ratio of average H2B-tdTomato signal to average nuclear NLS-GFP signal [*H2B/NLS*] is measured for each nucleus at every time point. If the difference in ratio between two consecutive time points [*Δ(H2B/NLS)*] exceeds 20% of the previous time point’s ratio, or if the *H2B/NLS* ratio increases continuously over the course of at least 5 consecutive time points, then NE rupture is determined to have begun (Fig 4A, red arrow). As the NE is repaired, NLS-GFP re-enters the nucleus, and *Δ(H2B/NLS)* becomes negative. The NE rupture event is completed when the *H2B/NLS* ratio returns close to its pre-rupture value and *Δ(H2B/NLS)* returns to zero (Fig 4C and 4D).

Since NE breakdown also occurs during mitosis, it is important to distinguish loss of nuclear NLS-GFP signals between mitotic cells and those exhibiting interphase NE rupture to avoid false positive detection of NE rupture. Mitosis and NE rupture both begin with NLS-GFP spreading into the cytoplasm and are initially indistinguishable from one another. However, during mitosis, two daughter nuclei form from one initial nucleus. Thus, if a nucleus is detected as undergoing NE rupture, and a new nucleus appears in its vicinity in the next time point, the event is reclassified as mitosis, and not NE rupture (S4 Fig).

### Manual verification

Automated data analysis can occasionally misidentify events; therefore, a video for manual verification is generated for every image sequence analyzed. The video is displayed on a graphical user interface, and the user can manually select individual nuclei and events to make corrections as necessary. Recorded data is exported to a file after manual validation.

## Automated analysis results

### Comparison of automated and manual analysis

To assess the accuracy of the program, we acquired two image sequences of BT-549 breast cancer cells migrating through a microfluidic device with 2-μm wide constrictions. The two image sequences were manually analyzed by four trained observers, recording constriction entry and exit times for each nucleus. One image sequence (S1 Video) was used to “train” the automated image analysis program to define the boundaries that mark entry and exit of the nucleus into/out of the constrictions. The program analyzed the video for six conditions, with constriction boundary lines placed either 5, 6, 7, 8, 9, or 10 μm from the constriction centerline. Comparing the program’s results with the manual results revealed that placing the boundaries 7 μm above and below the center produced the best agreement with the manual analysis.

**Fig 5:**
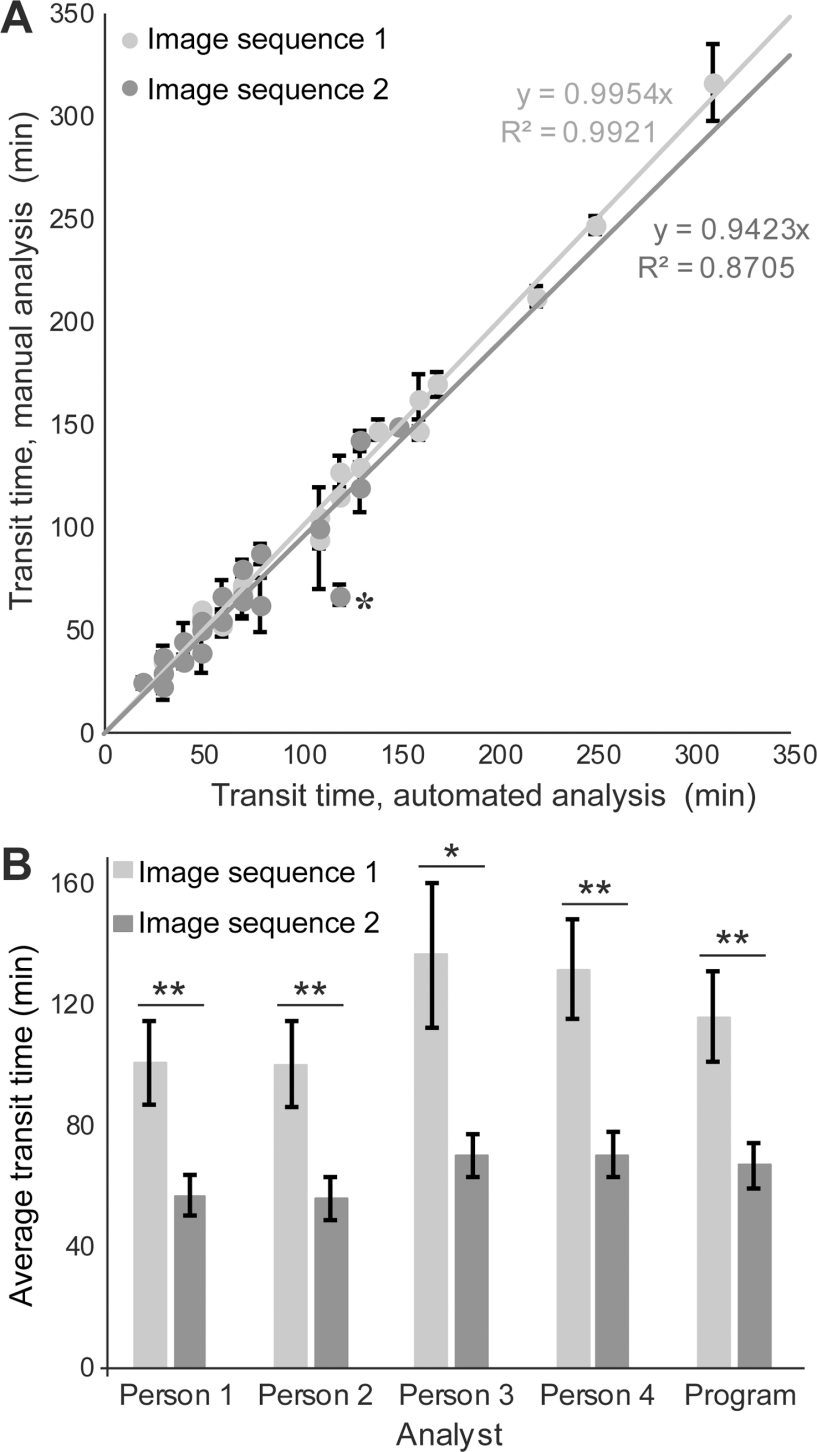
Verification of automated image analysis by comparison to manual analysis. (**A**) Automated analysis results plotted against manual analysis results (mean ± s.e.m. from four observers) for individual cells in two separate image sequences, each of which corresponds to a single section of a microfluidic device. For perfect agreement, the regression line plotted through these points would have a slope of one. Only one automated-analysis result substantially deviated from the manual reference, indicated by an asterisk. The manual analysis determined the nucleus to make two attempts to pass through the constriction, failing the first but succeeding the second time. The program identified this as a single, longer attempt. (**B**) Constriction transit times (mean ± s.e.m.) determined by four manual analysts and the automated analysis for the cells in the same two image sequences analyzed for panel A. Cells are BT-549 breast cancer and are either overexpressing lamin A (Image sequence 1) or an empty vector (Image sequence 2). Overexpression of lamin A results in less deformable nuclei and longer transit times through narrow constrictions (*, *p* < 0.05; **, *p* < 0.001 as calculated by ANOVA followed by Tukey’s multiple comparison test; *n* = 23–26 and 20–24 (depending on the analyst), respectively).

The other image sequence (S2 Video) was then used to “test” the program and the 7-μm constriction boundaries (Fig 5). Results of the automated image analysis for each nuclear transit event were compared to a manual analysis gold-standard, defined as the average result from four expert reviewers who assess nuclear transit times based on the visible deformation of the nucleus as it enters and exists the constriction. Comparison of the constriction transit times of individual cells determined by the program and four expert observers showed excellent agreement between the program and the manual gold-standard (Fig 5A). Similarly, the average constriction transit times computed by the program for each of the image sequences closely matched the data of the manual observers. Importantly, the program and all four observers correctly identified that cells that overexpress lamin A (Image sequence 1), and which have less deformable nuclei, had significantly longer transit times (*p* < 0.05) than mock-modified control cells (Image sequence 2) (Fig 5B).

Out of the 50 verified constriction transit events present and analyzed in the two image sequences, 46 were identified by the program prior to manual verification/correction, resulting in a “miss” rate of 8%. We recorded similar “miss” rates in six separate, independent image sequences analyzed by the program (data not shown). The four expert analysts had a combined “miss” rate of 10%, identifying an average of 45 events out of the total 50 transit events in the two image sequences. Therefore, even prior to manual verification, the program misses a similar or even lower number of nuclear constriction transit events than the expert analysts. The events missed by the program can be quickly and easily added to the programmatic analysis during the manual verification stage.

In addition to the identification of true nuclear transit events, person-to-person variability also applies to the transit times measured for each event. For any given transit event, the transit times recorded by the four manual analysts varied by greater than 2 image frames on average, ranging from 0 (complete agreement) to a maximum of 6 image frames. In all but one case (identified by the asterisk in Fig 5A) the program determined a transit time within the range of the manual measurements for that event. The single exception occurred in a cell that moved back and forth as it struggled to pass through the constriction, and the event could be identified as either one long attempt (as assessed by the expert analysts) or two shorter attempts (the first of which a failed attempt, followed by a successful attempt), as assessed by the program.

### Detection of differences in constriction transit times

**Fig 6:**
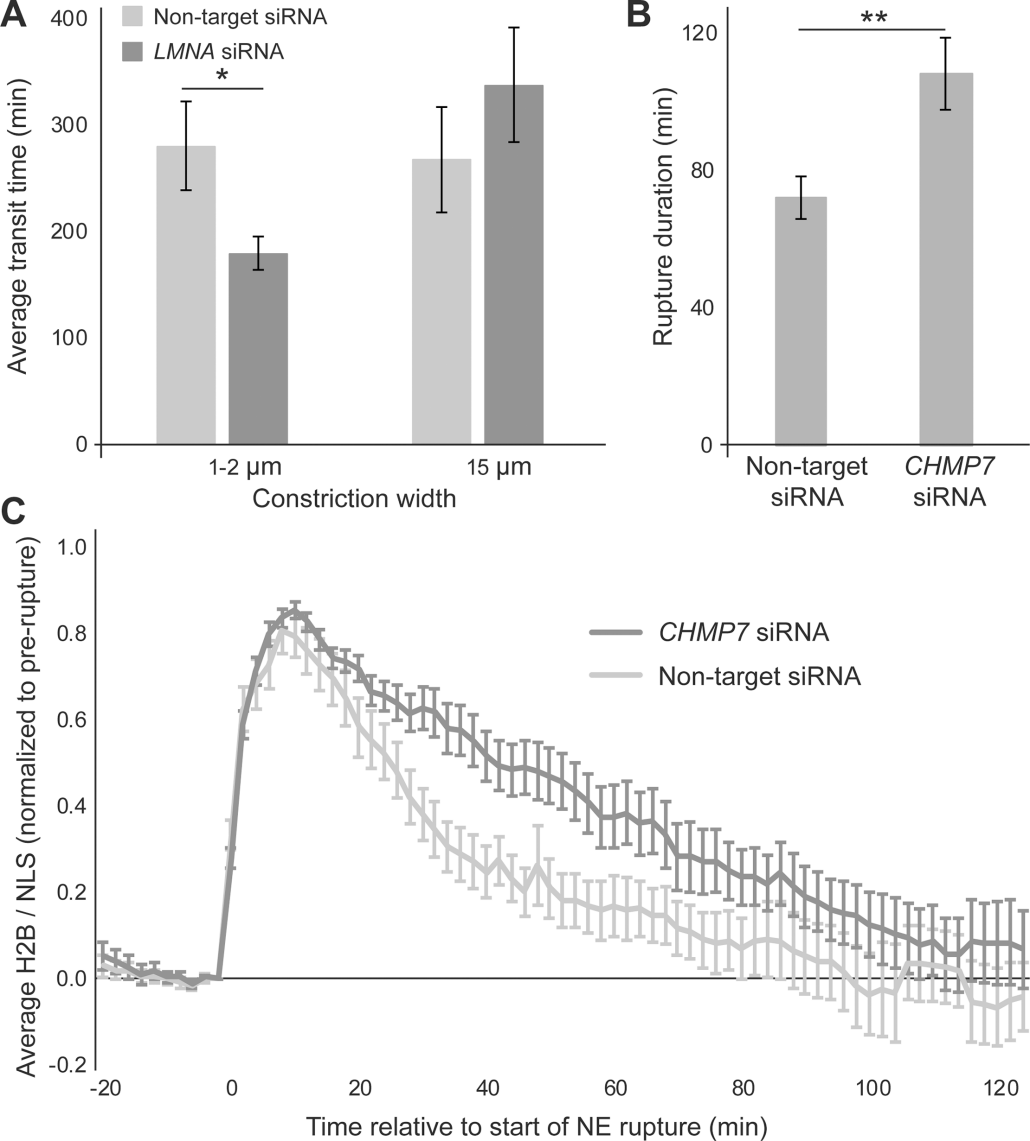
Application of automated image analysis program. (**A**) A549 cells depleted for lamin A/C (*n* = 40 cells across 4 microfluidic device sections) pass faster through small constrictions than non-target controls (*n* = 26 cells across 4 device sections). Transit times through larger openings were not statistically different (*, *p* < 0.05 as calculated by *t*-test; *n* = 21, 14, respectively). (**B**) HT-1080 cells depleted for CHMP7 (*n* = 65 cells across 3 device sections) took longer to repair their NE and restore nucleo-cytoplasmic compartmentalization than non-target controls (**, *p* < 0.01 as calculated by Mann-Whitney *t*-test; *n* = 48 cells across 3 device sections). (**C**) Dynamics of NE rupture and repair visualized by the ratio of nuclear H2B-tdTomato/NLS-GFP fluorescence for the CHMP7-depleted and non-target control cells. The H2B/NLS signal is expressed relative to its value at *t* = 0, i.e., immediately prior to rupture, and normalized to reach a peak value of 1 for each nucleus. CHMP7-depletion results in slower return to baseline, indicating delay in NE repair. Error bars represent mean ± s.e.m.

To assess whether the program could detect differences in constriction transit times in cell lines other than those used for the ‘training’ and ‘test’ data, we performed experiments with A549 human lung carcinoma cells treated with siRNA against lamin A/C or a non-target control (S5 Fig). A previous study found that lamin A/C depletion in A549 cells results in increased transit efficiency through small pores [6]. In another study, lamin A/C-deficient mouse embryo fibroblasts had significantly shorted transit times for passage through small constrictions than wild-type controls [7]. Consistent with the previous reports, the automated image analysis of our experiments found that lamin A/C-depleted cells passed faster through 1- and 2-μm wide constrictions than the non-target controls (*p* < 0.05). In contrast, both groups had comparable transit times (*p* = 0.34) when passing through 15-μm wide control channels that do not require nuclear deformation (Fig 6A).

### Detection of differences in NE rupture durations

The program’s ability to detect NE rupture events was verified through manual inspection of analyzed image sequences. To ensure that automated NE rupture detection is both precise and robust at identifying the durations of NE rupture/repair, we performed experiments with HT-1080 human fibrosarcoma cells treated with siRNA against the ESCRT-III family protein charged multivesicular body protein 7 (CHMP7) (S6 Fig). Since ESCRT-III proteins and CHMP7 are crucial for NE repair [16, 17, 29, 30], depletion of CHMP7 is expected to result in increased NE rupture duration. Automated image analysis confirmed that CHMP7-depleted HT-1080 cells experienced significantly longer NE rupture durations than the non-target controls (*p* < 0.01) (Fig 6B). CHMP7-depleted and non-target control cells showed similar H2B-tdTomato/NLS-GFP ratio values prior to NE rupture (*p* > 0.9) and 2 hours after rupture (*p* = 0.28), while CHMP7-depleted cells were slower to return to this baseline following NE rupture (Fig 6C).

## Discussion

We have developed and validated a MATLAB program for the automated and robust analysis of nuclear activity as cells migrate through microfluidic devices. This automation reduces the amount of time required to analyze an image sequence from multiple days/weeks to ~5 hours for a time-lapse experiment with 24 positions/experimental conditions and over 100 time points per position. Furthermore, the automated analysis removes person-to-person variability in the obtained results. The results produced by the program are in close agreement with expert manual analysis. The program is suitable for a broad range of applications that use microfluidic devices to study the migration of cells through confined environments, including analysis of transit times through pores of different size, or incidence of NE rupture. Previously, collecting data on a large number of cell lines, patient samples, or treatment conditions would have been impractical due to the substantial amount of time required to analyze the image sequences.

While the results presented here are based on a specific microfluidic migration device design, the modular nature of the program can be easily adapted to different design geometries, making it useful for a broad user base. Notably, the implemented automatic alignment and recognition of constrictions is independent of the constriction size and position. Similarly, fluorophores other than H2B-tdTomato and NLS-GFP can be used for the identification of nuclei and NE rupture, respectively.

The automated analysis is precise and robust enough to reach reliable conclusions concerning a population of cells’ constriction transit times and NE rupture durations with only minimal user supervision. Furthermore, the program can generate and collect data that would be challenging to obtain through manual analysis. For example, the ability to collect pixel intensity values in specific areas of interest allows the program to monitor the intensity of fluorescence in every nucleus over time. This allows the actual time course of NE repair to be observed, recorded, and compared across populations of cells. In contrast, while manual analysis can record the duration of NE rupture events, it lacks the accuracy required to analyze the extent of repair at earlier time points.

This nucleus tracking program is currently used only to monitor constriction transit times and NE rupture events, but could readily be expanded to a broader array of applications. For example, measurements of nuclear migration persistence, i.e., the tendency of the nucleus to move in a constant direction, could be recorded, since nucleus centroids are determined for each time point. Such an analysis would be extremely tedious and highly time-consuming if done manually. The automated analysis could also be expanded to include cell death detection, for example, based on the permanent loss of NLS-GFP intensity and unmatched nuclei after cell death. Automated cell death analysis could be useful for screening of drugs that target metastatic cancer cells. Under the conditions used in the current experiments, only few cells died during imaging, and these cases appeared to be the result of confined migration and continuous NE rupture, rather than phototoxicity, consistent with previous reports [13, 16]. Additionally, the object identification and tracking elements of the program could be applied to any other set of time-lapse images, for example, to cells migrating on 2-D substrates or contact-printed micropatterns.

## Materials and methods

### Creating microfluidic devices

Microfluidic devices were created from a silicon wafer mold fabricated by 2-layer SU-8 photolithography as described previously [20]. Polydimethylsiloxane (PDMS) was created by mixing Sylgard 184 Silicone Elastomer Base and Silicone Elastomer Curing Agent in a 10:1 ratio as per the manufacturer’s instructions (Corning). A vacuum chamber was then used to remove air bubbles, and the PDMS was poured into the silicon mold and baked for 2 hours at 65°C to solidify. After removal from the mold, the PDMS was cut to size, and biopsy needles were used to cut out device reservoirs and perfusion channel inlets/outlets.

Devices and glass slides were then washed with deionized water and isopropyl alcohol, dried, and plasma cleaned for five minutes. Covalent bonding of the devices to the slides then occurred by gentle pressing of the device onto the slide and placing the slide on a hot plate at 95°C for 5 minutes. Slide-bound devices were then brought to a tissue-culture hood, and rinsed with 70% ethanol followed by deionized water. Device reservoirs were filled with 20 μg/mL of fibronectin in PBS (for A549 and BT-549 cells) or 0.05 mg/mL of collagen in 0.02 M acetic acid (HT-1080 cells). Devices were kept in a sealed Petri dish at 4°C overnight to allow binding of the protein to the glass slide and PDMS.

### Cell culture

The human lung carcinoma cell line A549 (ATCC) was cultured in F-12K media (Gibco) supplemented with 10% fetal bovine serum (FBS, VWR) and 1% penicillin and streptomycin (pen/strep, Gibco). The human fibrosarcoma cell line HT-1080 (ATCC) was cultured in DMEM supplemented with 10% FBS and 1% pen/strep. BT-549 breast cancer cells (ATCC) were cultured in RPMI media supplemented with 10% FBS and 1% pen/strep. All cells were cultured at 37°C and 5% CO_2_.

### Generation of fluorescently labelled cell lines

Cell lines were stably modified with a retroviral vector to express both the NE rupture reporter NLS-GFP, and histone marker H2B-tdTomato (pQCXIP-NLS-copGFP-P2A-H2B-tdTomato-IRES-puro, System Biosciences). The retroviral vector was generated in two steps. LifeAct-GFP was digested out of the pQCXIP-LifeAct-GFP-P2A-H2B-tdTomato vector, and NLS-copGFP was ligated into the vector. NLS-copGFP was obtained from a lentiviral vector (pCDH-CMV-NLS-copGFP-EF1-blastiS) via digestion. The product was then amplified via touchdown PCR, introducing the NotI and AgeI restriction sites, using the following forward and reverse primers, respectively: 5’-CAAGCGGCCGCACCATGACTGCTCCAAAGAAGAAGCG-3’ and 5'-GCAACCGGTGCGAGATCCGGTGGAGCCGG-3'. Retroviral particles were produced via 293-GPG cell transfection with the plasmid and Lipofectamine 2000 (Invitrogen) following the manufacturer’s protocol. Retrovirus-containing supernatants were collected once every 24 hours for 5 days following transfection and strained through a 0.22 μm filter. Cells were seeded into 6-well plates to reach 50–60% confluency on the day of infection, and were transduced with viral stock in the presence of 8 μg/mL polybrene (Sigma-Aldrich) every 24 hours for three days. On the fourth day, the viral solution was replaced with fresh culture medium, and cells were cultured for three days before selection with puromycin. After selection, cells were sorted on a BD FACSARIA FUSION fluorescence activated cell sorter (Cornell University Biotechnology Resource Center), and used for experiments or frozen down.

### siRNA mediated depletion of lamin A/C and CHMP7

Lamin A/C depletion in A549 cells and CHMP7 depletion in HT-1080 cells was accomplished using DharmaFECT (Dharmacon) and target-specific siRNA according to the manufacturer’s protocol, with final siRNA concentrations of 2.5 nM (*LMNA*) and 100 nM (*CHMP7*). SmartPool siRNA oligonucleotides, containing four target sequences in one mix to reduce off-target effects, were purchased from Dharmacon (GE Healthcare): human LMNA (ON-TARGET plus SMART pool, L-004978-00), human CHMP7 (ON-TARGETplus SMARTpool, L-015514-01), and non-targeting control siRNA (ON-TARGETplus non-targeting pool, D-001810-10).

### Generation of cell lines overexpressing lamin A

BT-549 cells were stably modified to express NLS-RFP using a pCDH lentiviral construct (Systems Biosciences). After selection with blasticidin (InvivoGen), cells were modified with a retroviral bicistronic constructs expressing lamin A or a mock control as described previously [31]. Cells were then sorted for RFP- and GFP-expressing cells before being used in experiments.

### Seeding cells into microfluidic devices

Cells were trypsynized, centrifuged, counted and resuspended in media to a concentration of 5,000 cells/μL. A cell suspension containing 30,000 cells was added to the inlet port of each device. Device reservoirs were then filled with media and kept in an incubator overnight to allow cell attachment to the fibronectin- or collagen-coated devices. In the morning media was removed, and one reservoir was filled with plain media while the other was simultaneously filled with FBS-supplemented media, creating a chemotactic gradient to promote cell migration across the constrictions. Devices were then returned to the incubator until the start of imaging. Just prior to imaging, media was again removed from the devices. The FBS gradient was established in the same manner as before, but with Fluorobrite (Gibco) imaging media containing 25 mM 4-(2-hydroxyethyl)-1-piperazineethanesulfonic acid (HEPES, Gibco) to keep the cells at physiologic pH in the absence of 5% CO_2_ during time-lapse imaging. Cover slips were placed over the devices to prevent evaporation of media.

A detailed schematic of the microfluidic device used, including reservoirs, cell seeding ports, and constrictions, can be found elsewhere [20]. The constriction design we used here is the rightmost one presented in Fig 1G of that reference. Designs for the device are available at: http://lammerding.wicmb.cornell.edu/migration-device-design/

### Imaging

Migration devices were imaged on an inverted Zeiss Observer Z1 microscope with a temperature-controlled stage set to 37°C at 20× magnification (NA 0.8 air objective) and a CoolSNAP EZ CCD camera (Photometrics). Zen software (Zeiss) was used to automate image acquisition, taking images of specific sections of the migration device every 2 min (HT-1080) or 10 min (A549 and BT-549). Images of the migration device and cells were acquired with differential interference contrast (DIC); fluorescence microscopy was used to capture the NLS-GFP signal (excitation with 450–490 nm light, collection of emission at 500–550 nm; exposure time of 75 ms), and the H2B-tdTomato signal (excitation by 550–580 nm light, collection of 590–650 nm light; exposure time of 400 ms). All images were saved in the Carl Zeiss Image (*.czi) format.

### Western blotting

To quantify protein depletion, Western blots were performed with cell lysates. Parallel prepared samples of the siRNA treated cells were lysed at the time of imaging in high salt radioimmunoprecipitation assay (HS RIPA) buffer. Lysates were vortexed for five minutes to shear DNA, heated to 93°C for two minutes, and centrifuged to pellet DNA. Protein concentrations were determined by Bradford assay. Equal amounts of protein (15 μg) for each sample were loaded into a NuPAGE 10% Bis-Tris gel (Gibco). Gels ran in 3-(N-morpholino)propanesulfonic acid (MOPS) buffer at 100 V until the ladder bands began to separate, and then at 175 V until completion. Proteins were then transferred to a polyvinylidene difluoride (PVDF, Millipore) membrane at 16 V for one hour. Gels were stained with Coomassie to observe quality of protein loading. After transfer, the membrane was blocked for at least one hour at room temperature in 5% milk. Primary antibodies (anti-lamin A/C, Santa Cruz, sc-6215, dilution 1:2000; anti-CHMP7, Sigma, HPA036119, dilution 1:200; anti-actin, Santa Cruz, sc-1615 HRP, dilution 1:2000) were added and left on overnight at 4°C. Secondary antibodies (donkey anti-rabbit 800 cw and donkey anti-mouse 680 RD, Licor) were incubated for one hour at room temperature prior to imaging on a LI-COR Odyssey CLx.

### Image analysis

Image sequences were analyzed using the custom-written MATLAB program. To allow the program to read the images in the .czi format, the Bio-Formats package was downloaded from the Open Microscopy Environment’s webpage and added to the MATLAB search path. Constriction passage times as well as NE rupture events were recorded for all cells. Data was automatically exported into a comma-separated values (*.csv) file for use with Microsoft Excel. To verify the accuracy of the program, results for selected image sequences were compared to results from manual analysis using Zen software.

### Statistical analysis

Statistical analysis was performed on GraphPad’s Prism software. Distributions of constriction transit times and NE rupture durations were tested for normality, and their means were compared using the appropriate statistical tests. Two-tailed t-tests with Welch’s correction for unequal variances were used to compare two normally distributed means. The Mann-Whitney test was used to compare two means if either was not normally distributed. One-way analysis of variance (ANOVA) followed by Tukey’s multiple comparison test was used to compare the constriction transit times determined by the program with those determined through manual analysis. In the comparison between manual and automated image analysis, three data points were excluded due to large discrepancies in the manual analysis between the four observers. Two-tailed t-tests with the Bonferroni correction were used to compare the normalized fluorescent intensities during NE rupture events.

## Supporting information

S1 Fig Automated detection of microfluidic constrictions. (**A**) Image of a microfluidic device section prior to rotation. The rows of constrictions are angled upwards. (**B**) The results of a circular Hough transform have been superimposed onto the original image (red). These circles represent the columns that form the constrictions and their centers are used to determine the angle of rotation. (**C**) Image of the microfluidic device rotated so that the rows of constrictions are horizontal. (**D**) A circular Hough transform is applied to the rotated image (red). Now the locations of the circles’ centers can be used to place boundaries for entering and exiting a constriction (blue).
(TIF)

S2 Fig Example of watershed segmentation. **1)** An image of migrating nuclei is binarized based on the H2B-tdTomato signal. Since the two nuclei shown are touching, they become a single object in the binary image. **2)** The binary image is inverted so that the distance transform, which measures the distance from any given pixel to the nearest non-zero (white) pixel, works as needed. **3)** The distance transform is applied. Inside of the nuclei, values are higher (closer to white in the image) the farther they are from the nucleus’ nearest edge. **4)** The image from the distance transform is inverted so that the watershed segmentation works as needed. Now the center of a nucleus is a minimum (closer to black in the image). **5a)** A watershed transform is applied. The black lines represent watershed lines, cutting through local maxima to separate all of the image’s local minima or catchment basins (each of which is shown as a different shade of gray). **6a)** The watershed lines are used to segment the original binary image. Over-segmentation has occurred since the top nucleus has been erroneously split into three separate objects. **5b)** An h-minima transform is applied to the inversion of the distance transform. Local minima that are too shallow are removed from the image to prevent over-segmentation from occurring. **6b)** A watershed transform is applied. Since negligible minima were removed from the image there are now only two catchment basins. **7b)** The watershed line is used to segment the original binary image. Application of the h-minima transform during this process prevented over-segmentation from occurring. Use of the watershed segmentation successfully separated two touching nuclei into distinct objects.
(TIF)

S3 Fig Examples on constriction transit identification. **A)** The two nuclei depicted are identified as attempting to pass through the constrictions by the program. This is because the leading (top) edges of their bounding boxes (shown in blue) are above the lower constriction boundary (both constriction boundaries depicted as dashed green lines), but their bounding box trailing (lower) edges are still below the upper constriction boundary. **B)** Movement of the nucleus as shown here would result in the program recording a successful constriction passage since the trailing edge of the nucleus’ bounding box eventually crosses the upper constriction boundary. **C)** Movement of the nucleus as shown here would result in the program recording a failed constriction passage since the leading edge of the nucleus’ bounding box recedes below the lower constriction boundary.
(TIF)

S4 Fig Detection of mitotic cells to reduce misclassification of nuclear envelope rupture and incorrect nucleus matching. (A) Example of incorrectly labeled nuclear envelope rupture (box with the letter R) and unmatched nucleus appearing in the fourth frame (magenta box) when a mitotic cell divides into two daughter cells. (B) Results obtained with the automated mitosis detection feature of the program. The nucleus outlined in cyan is now recorded as undergoing division (signified by the letter D). The nuclei outlined in magenta and gray are recorded as daughters of the cyan-outlined nucleus. Proper distinction between mitosis and nuclear envelope rupture is necessary to prevent recording of false positive nuclear envelope rupture data.
(TIF)

S5 Fig Depletion of lamin A/C by siRNA. (**A**) Western blot of the A549 cells used in four independent migration experiments. Visual inspection reveals lower lamin A/C expression in the cells that received the knockdown (KD) as compared to the cells that received the non-targeting siRNA (NT). (**B**) Quantification of lamin A levels, normalized to actin loading control. (**C**) Quantification of lamin C levels, normalized to actin loading control. *, *p* < 0.05
(TIF)

S6 Fig Depletion of CHMP7 by siRNA. Western blot of the HT1080 cells used in three independent migration experiments. Visual inspection confirms lower CHMP7 expression in the cells that received the knockdown (KD) as compared to the cells that received the non-targeting siRNA (NT). (**B**) Quantification of CHMP7 levels, normalized to actin loading control. ***, *p* < 0.001.
(TIF)

S1 Video Video of migrating nuclei corresponding to Image Sequence 1 of Fig 5. Video of BT-549 cells migrating through a microfluidic device with 2-μm wide constrictions, corresponding to Image Sequence 1 in Fig 5. Cells were modified to express NLS-GFP and over-express lamin A, resulting in more rigid nuclei and impaired transit through the constrictions. This video was used as the “training” data for the program.
(AVI)

S2 Video Video of migrating nuclei corresponding to Image Sequence 2 of Fig 5. Video of BT-549 cells migrating through a microfluidic device with 2-μm wide constrictions, corresponding to Image Sequence 2 in Fig 5. Cells were modified to express NLS-GFP and a mock control vector. This video was used as the “test” data for the program.
(AVI)
