# Supplementary figures and images for: Automated analysis of cell migration and nuclear envelope rupture in confined environments
Source: PLoS One. 2018 Apr 12;13(4):e0195664. doi: 10.1371/journal.pone.0195664 (PMC5896979; doi:10.1371/journal.pone.0195664)

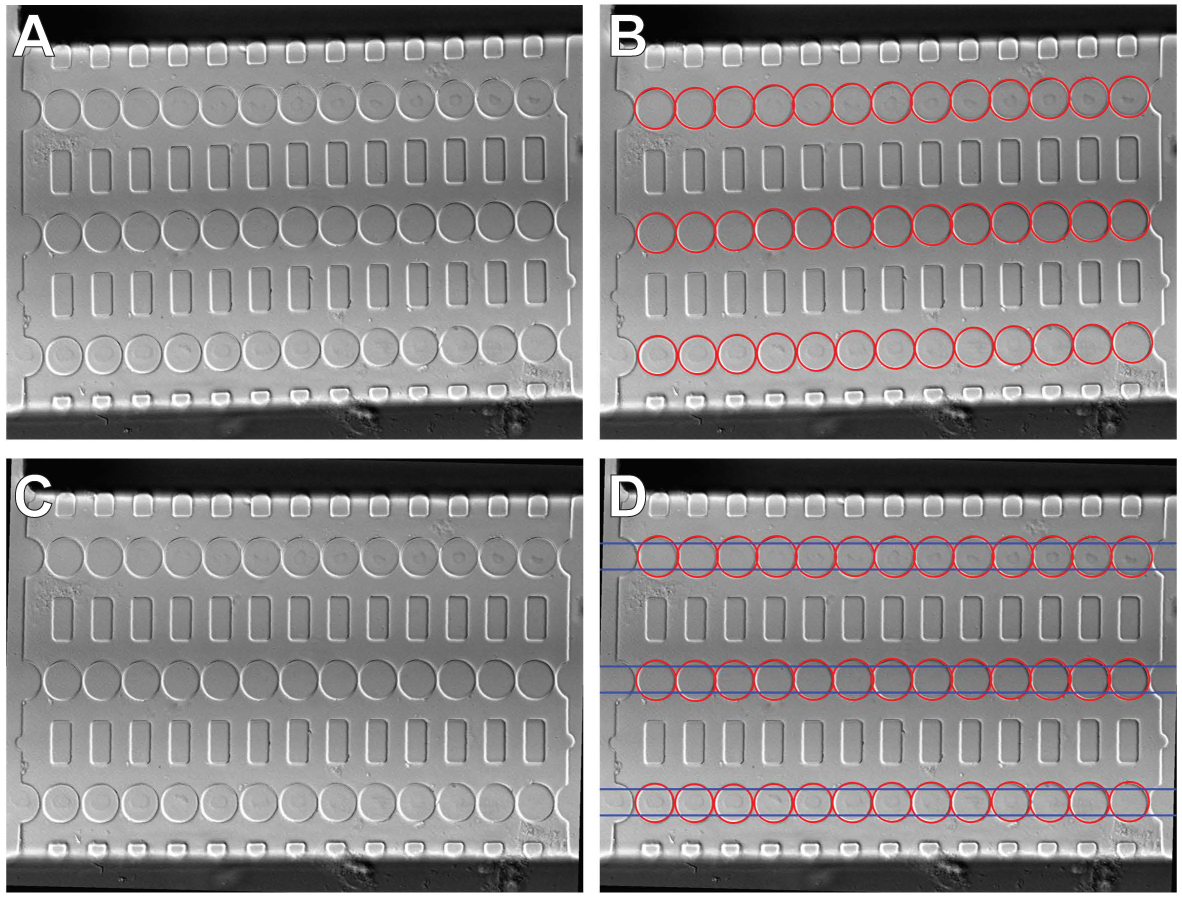

Supplement: S1 Fig — (A) Image of a microfluidic device section prior to rotation. The rows of constrictions are angled upwards. (B) The results of a circular Hough transform have been superimposed onto the original image (red). These circles represent the columns that form the constrictions and their centers are used to determine the angle of rotation. (C) Image of the microfluidic device rotated so that the rows of constrictions are horizontal. (D) A circular Hough transform is applied to the rotated image (red). Now the locations of the circles’ centers can be used to place boundaries for entering and exiting a constriction (blue). (TIF) [file pone.0195664.s001.tif]

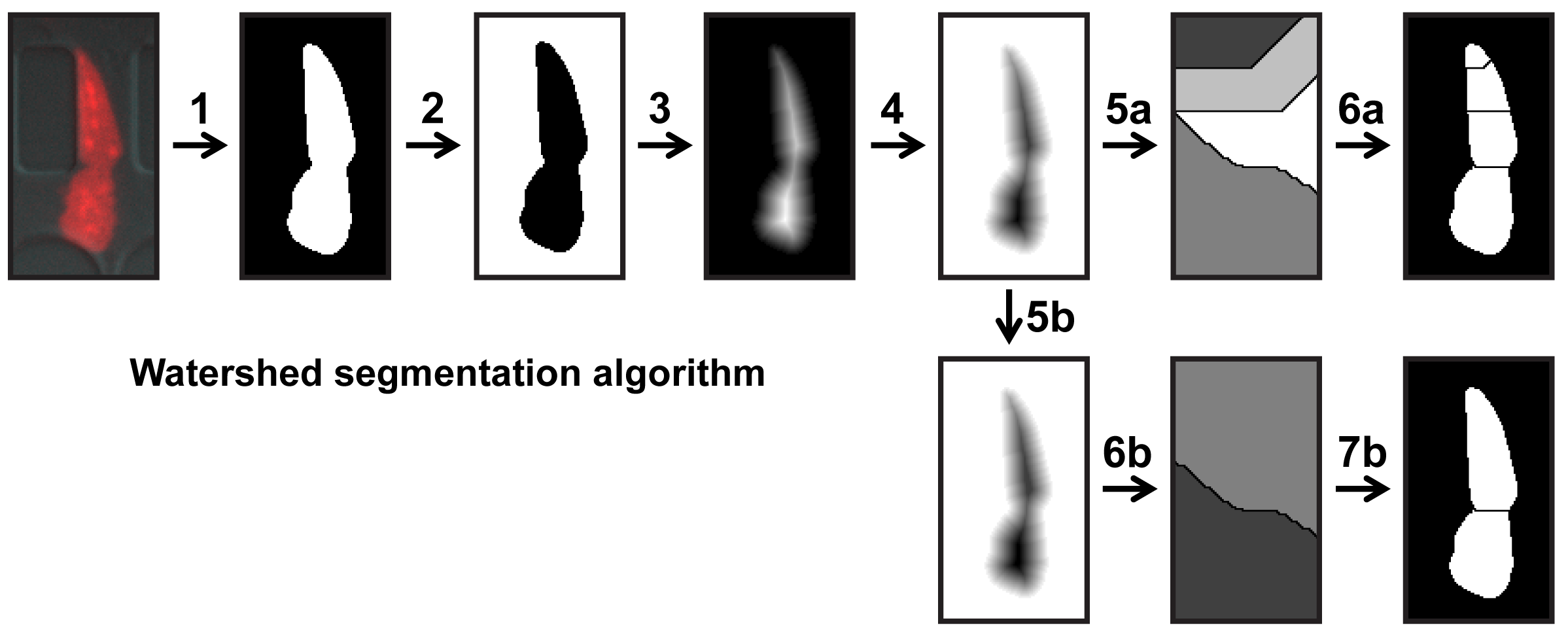

Supplement: S2 Fig — 1) An image of migrating nuclei is binarized based on the H2B-tdTomato signal. Since the two nuclei shown are touching, they become a single object in the binary image. 2) The binary image is inverted so that the distance transform, which measures the distance from any given pixel to the nearest non-zero (white) pixel, works as needed. 3) The distance transform is applied. Inside of the nuclei, values are higher (closer to white in the image) the farther they are from the nucleus’ nearest edge. 4) The image from the distance transform is inverted so that the watershed segmentation works as needed. Now the center of a nucleus is a minimum (closer to black in the image). 5a) A watershed transform is applied. The black lines represent watershed lines, cutting through local maxima to separate all of the image’s local minima or catchment basins (each of which is shown as a different shade of gray). 6a) The watershed lines are used to segment the original binary image. Over-segmentation has occurred since the top nucleus has been erroneously split into three separate objects. 5b) An h-minima transform is applied to the inversion of the distance transform. Local minima that are too shallow are removed from the image to prevent over-segmentation from occurring. 6b) A watershed transform is applied. Since negligible minima were removed from the image there are now only two catchment basins. 7b) The watershed line is used to segment the original binary image. Application of the h-minima transform during this process prevented over-segmentation from occurring. Use of the watershed segmentation successfully separated two touching nuclei into distinct objects. (TIF) [file pone.0195664.s002.tif]

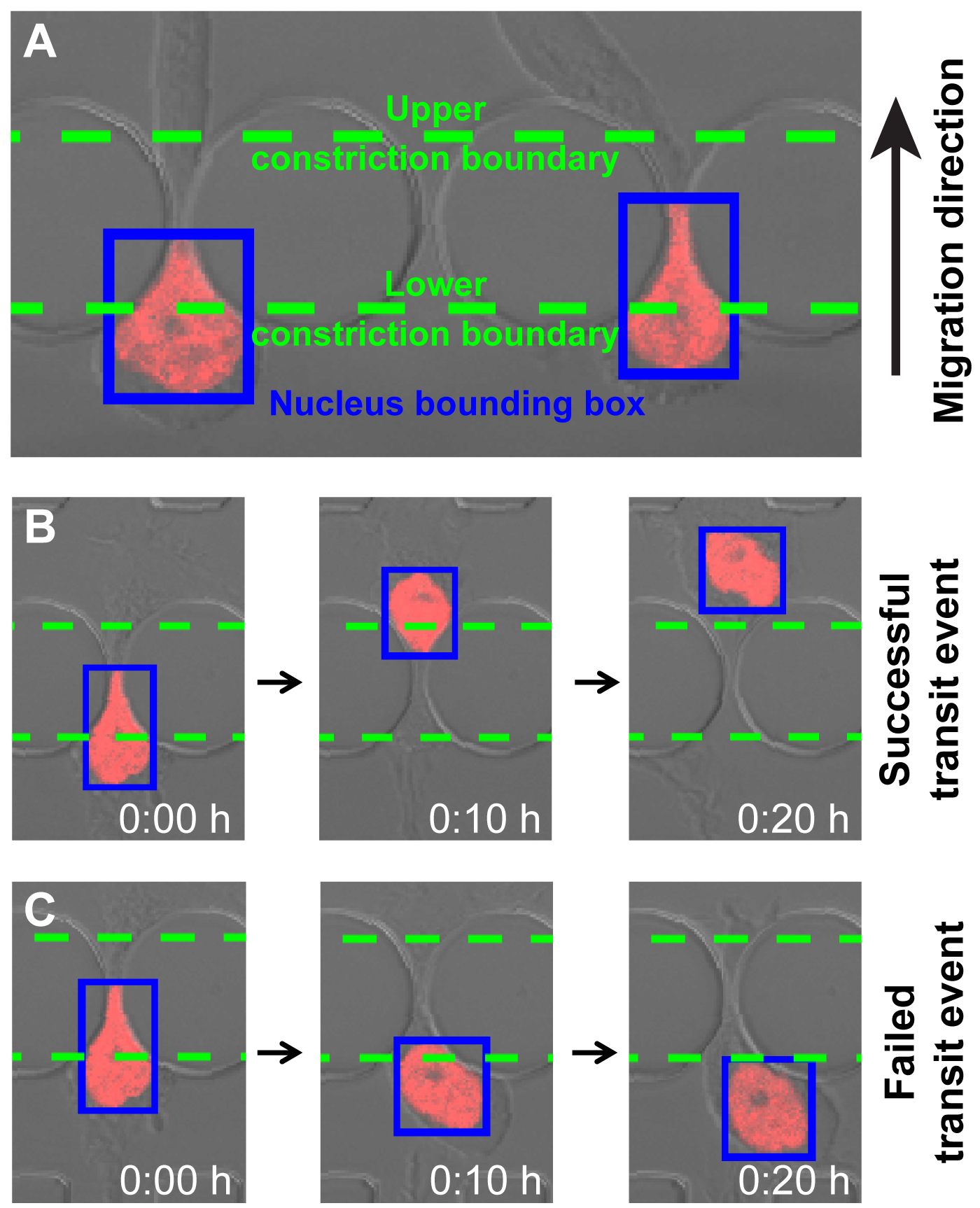

Supplement: S3 Fig — A) The two nuclei depicted are identified as attempting to pass through the constrictions by the program. This is because the leading (top) edges of their bounding boxes (shown in blue) are above the lower constriction boundary (both constriction boundaries depicted as dashed green lines), but their bounding box trailing (lower) edges are still below the upper constriction boundary. B) Movement of the nucleus as shown here would result in the program recording a successful constriction passage since the trailing edge of the nucleus’ bounding box eventually crosses the upper constriction boundary. C) Movement of the nucleus as shown here would result in the program recording a failed constriction passage since the leading edge of the nucleus’ bounding box recedes below the lower constriction boundary. (TIF) [file pone.0195664.s003.tif]

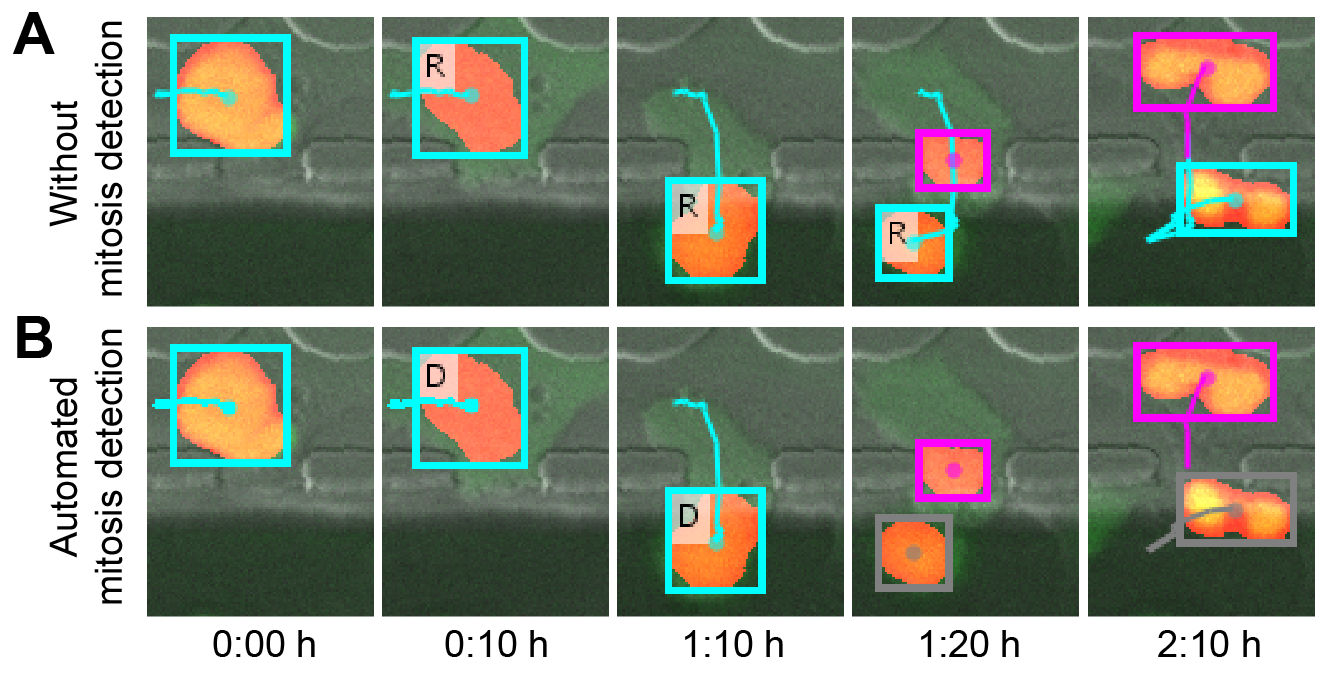

Supplement: S4 Fig — (A) Example of incorrectly labeled nuclear envelope rupture (box with the letter R) and unmatched nucleus appearing in the fourth frame (magenta box) when a mitotic cell divides into two daughter cells. (B) Results obtained with the automated mitosis detection feature of the program. The nucleus outlined in cyan is now recorded as undergoing division (signified by the letter D). The nuclei outlined in magenta and gray are recorded as daughters of the cyan-outlined nucleus. Proper distinction between mitosis and nuclear envelope rupture is necessary to prevent recording of false positive nuclear envelope rupture data. (TIF) [file pone.0195664.s004.tif]

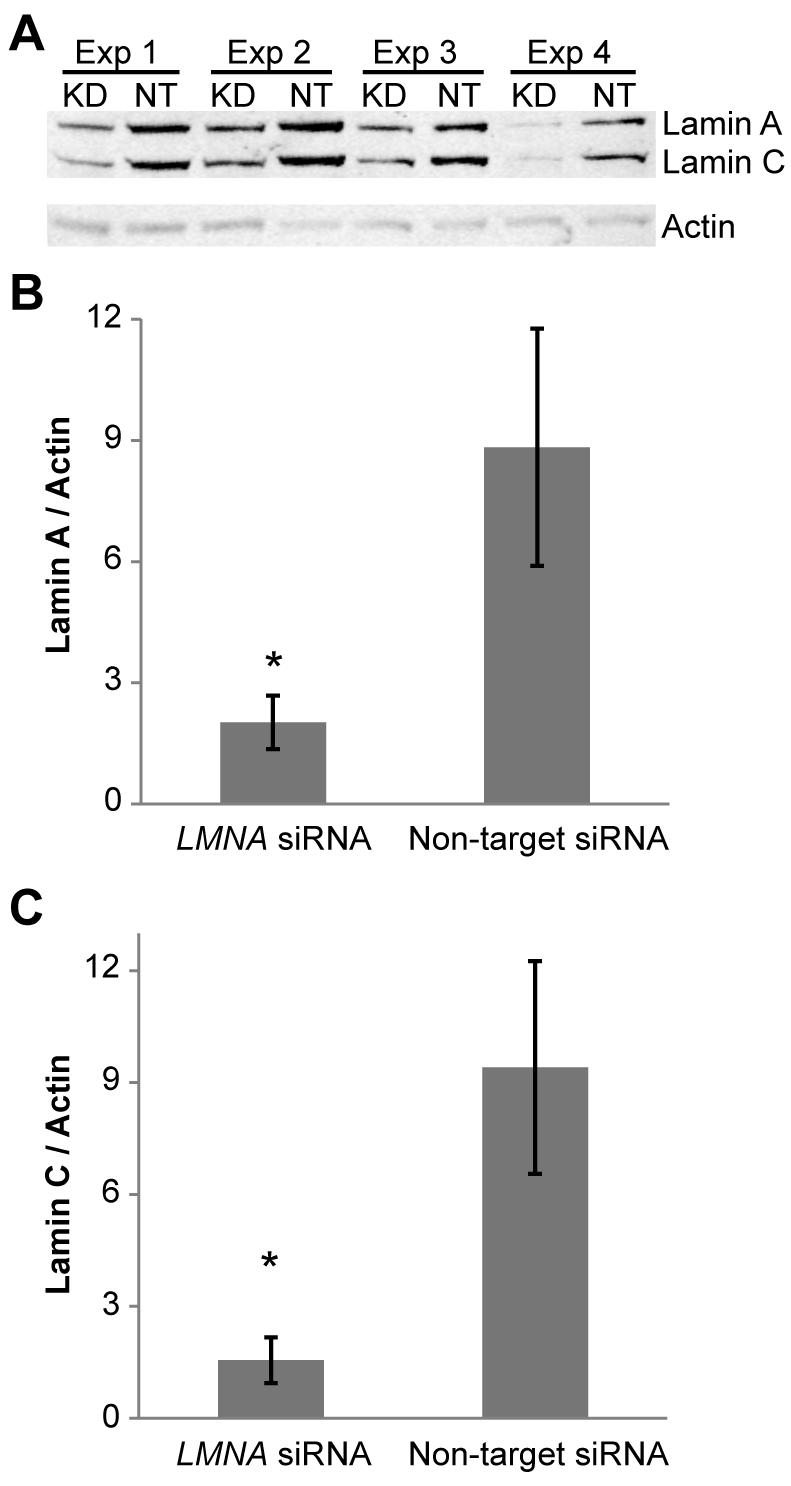

Supplement: S5 Fig — (A) Western blot of the A549 cells used in four independent migration experiments. Visual inspection reveals lower lamin A/C expression in the cells that received the knockdown (KD) as compared to the cells that received the non-targeting siRNA (NT). (B) Quantification of lamin A levels, normalized to actin loading control. (C) Quantification of lamin C levels, normalized to actin loading control. *, p < 0.05 (TIF) [file pone.0195664.s005.tif]

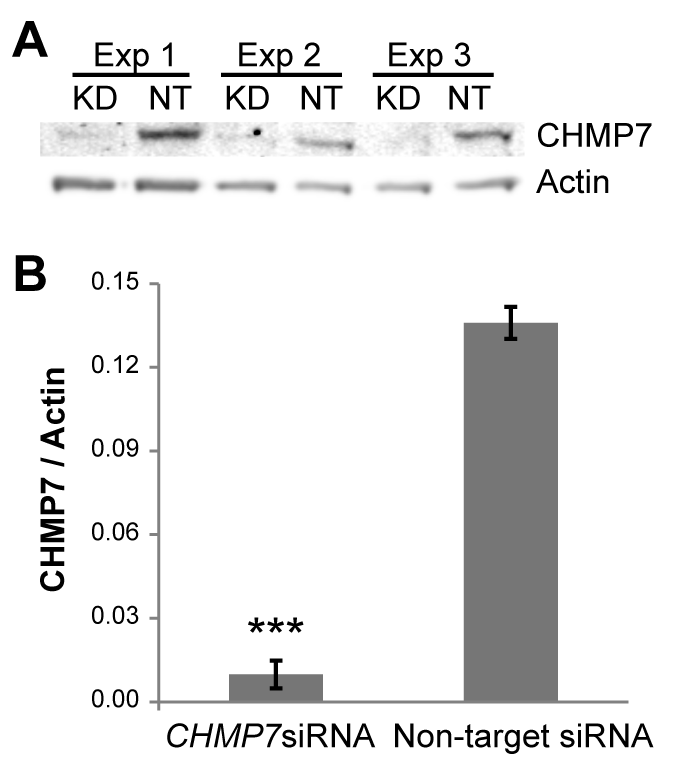

Supplement: S6 Fig — Western blot of the HT1080 cells used in three independent migration experiments. Visual inspection confirms lower CHMP7 expression in the cells that received the knockdown (KD) as compared to the cells that received the non-targeting siRNA (NT). (B) Quantification of CHMP7 levels, normalized to actin loading control. ***, p < 0.001. (TIF) [file pone.0195664.s006.tif]
